# Ultrathin Bronchoscopy Without Virtual Navigation for Diagnosis of Peripheral Lung Lesions

**DOI:** 10.1007/s00408-024-00695-1

**Published:** 2024-06-12

**Authors:** Ali Sadoughi, Shwe Synn, Christine Chan, David Schecter, Gabriel Hernandez Romero, Sahil Virdi, Abhishek Sarkar, Mimi Kim

**Affiliations:** 1 Division of Pulmonary, Montefiore Medical Center, Albert Einstein College of Medicine, New York City, USA; 2 Department of Medicine, Montefiore Medical Center, Albert Einstein College of Medicine, New York City, USA; 3 Department of Medicine, Jacobi Medical Center, Albert Einstein College of Medicine, Bronx, USA; 4 Division of pulmonary and critical care, United Hospital Center, West Virginia University Health System, Charleston, USA; 5 Section of Interventional Pulmonology, Department of Pulmonary, Critical Care, and Sleep Medicine, Westchester Medical Center / New York Medical College, Valhalla, USA; 6 Division of Biostatistics, Department of Epidemiology & Population Health, Albert Einstein College of Medicine, Bronx, USA

**Keywords:** Ultrathin bronchoscopy, Lung nodule, Peripheral airway bronchoscopy, Radial endobronchial ultrasound, Lung cancer

## Abstract

**Background:**

The increasing incidence of encountering lung nodules necessitates an ongoing search for improved diagnostic procedures. Various bronchoscopic technologies have been introduced or are in development, but further studies are needed to define a method that fits best in clinical practice and health care systems.

**Research question:**

How do basic bronchoscopic tools including a combination of thin (outer diameter 4.2 mm) and ultrathin bronchoscopes (outer diameter 3.0 mm), radial endobronchial ultrasound (rEBUS) and fluoroscopy perform in peripheral pulmonary lesion diagnosis?

**Study Design and Methods:**

This is a retrospective review of the performance of peripheral bronchoscopy using thin and ultrathin bronchoscopy with rEBUS and 2D fluoroscopy without a navigational system for evaluating peripheral lung lesions in a single academic medical center from 11/2015 to 1/2021. We used a strict definition for diagnostic yield and assessed the impact of different variables on diagnostic yield, specifically after employment of the ultrathin bronchoscope. Logistic regression models were employed to assess the independent associations of the most impactful variables.

**Results:**

A total of 322 patients were included in this study. The median of the long axis diameter was 2.2 cm and the median distance of the center of the lesion from the visceral pleural surface was 1.9 cm. Overall diagnostic yield was 81.3% after employment of the ultrathin bronchoscope, with more detection of concentric rEBUS views (93% vs. 78%, *p* < 0.001). Sensitivity for detecting malignancy also increased from 60.5% to 74.7% (*p* = 0.033) after incorporating the ultrathin scope into practice, while bronchus sign and peripheral location of the lesion were not found to affect diagnostic yield. Concentric rEBUS view, solid appearance, upper/middle lobe location and larger size of the nodules were found to be independent predictors of successful achievement of diagnosis at bronchoscopy.

Interpretation: This study demonstrates a high diagnostic yield of biopsy of lung lesions achieved by utilization of thin and ultrathin bronchoscopes. Direct visualization of small peripheral airways with simultaneous rEBUS confirmation increased localization rate of small lesions in a conventional bronchoscopy setting without virtual navigational planning.

## Introduction

The American Cancer Society estimated new cases of lung cancer in 2023 to be more than 238,000. While lung cancer incidence and mortality has steadily decreased over recent years, there are still approximately 350 persons dying each day from lung cancer in the United States [1]. In 2010, more than 4.8 million Americans had at least one chest CT scan, with 1.57 million having a lung nodule identified on at least one of those scans [2]. With implementation of low-dose CT lung cancer screening, an increasing number of lung nodules are expected to be discovered. Although results of the National Lung Screening Trial showed the benefits of low-dose helical CT scans, most notably a 20% decrease in lung cancer deaths, the downside of this comprehensive screening was a high rate of false-positive nodules (95%) adding to the need for precise diagnostic technology [3].

Diagnosis of lung nodules remains challenging and may delay the care of patients with lung cancer. With increased detection of lung nodules, development of minimally invasive and at the same time affordable techniques for detection of lung cancer in early stages remains a priority. Recommendations from the American College of Chest Physicians (ACCP) evidence-based guidelines for the management of pulmonary lesions begin with estimating the probability of cancer, and further evaluation including biopsy for those patients with lesions of intermediate probability (5–65%) for malignancy [4, 5]. CT-guided biopsy via the chest wall or surgical biopsy were traditionally used as the most reliable techniques with higher diagnostic yield. Both methods are associated with risks and discomfort for patients [6, 7]. Bronchoscopy has lower complication rates, but not every bronchoscopic method provides high diagnostic yield. High quality comparative studies of diagnostic yield and the associated costs are lacking. A recently published meta-analysis reported that despite advances in bronchoscopic technology to diagnose peripheral lung lesions, the diagnostic yield of guided bronchoscopy has not improved [8]. There is wide disparity in diagnostic yield among bronchoscopists and the various technologies used. Several technologies have evolved which allow clinicians to reach pulmonary lesions via bronchoscopy, such as electromagnetic navigation (ENB), virtual bronchoscopy (VB), radial endobronchial ultrasound (r-EBUS) with or without guide sheath, ultrathin bronchoscopy, robot assisted bronchoscopy, and cone-beam computed tomography (CBCT) guided bronchoscopy. The use of rEBUS allows confirmation of a lesion outside the tracheobronchial tree. The other tool that can confirm the localization of the target during bronchoscopy is CBCT. CBCT may help re-route the bronchoscope in situations where navigation into the targeted lesion fails. More recently advanced bronchoscopy platforms combined with 3D imaging or augmented fluoroscopy to overcome some of the challenges in bronchoscopy [9–26]. The high costs associated with the acquisition of some of these technologies and the cost of accessory tools are limiting factors for universal clinical adoption.

The ultrathin bronchoscope with outer diameter (OD) at tip 3.0 mm and working channel diameter (WC) 1.7 mm can reach more distal bronchi compared to other traditional bronchoscopes and provide an unprecedented visualization of the small airways. Studies have shown that its addition to the conventional bronchoscopy setting combined with rEBUS, with or without navigational platforms, has increased diagnostic yield of peripheral pulmonary lesions [27–31]. Despite these promising results and the relatively low cost of the procedure, it remains underutilized in clinical practice. In this study, we sought to evaluate the performance of the basic bronchoscopic tools including combination of thin and ultrathin bronchoscopes, radial endobronchial ultrasound (rEBUS), and fluoroscopy in the diagnosis of peripheral pulmonary lesions. We assessed the impact of different variables including patient characteristics, nodule size and location, and bronchoscopic findings on diagnostic yield. We analyzed independent associations of the most impactful variables and described potential predictors of a successful diagnostic bronchoscopy.

## Methods

We performed a retrospective review of peripheral bronchoscopy using thin and ultrathin bronchoscopy with rEBUS and 2D fluoroscopy without any navigational system for peripheral lung lesions in our academic medical center from November 2015 through January 2021. The practice pattern was noticeably affected by the acquisition of an ultrathin bronchoscope (Olympus BF-MP190 with OD at tip 3.0 mm, WC 1.7 mm) on 3/4/2019 while the thin bronchoscope Olympus BF-P190 (OD 4.2 mm, WC 2.0 mm) continued to be used. We assessed diagnostic yield and the impact of different variables on outcomes. We compared the results between two groups of patients before and after employment of the ultrathin bronchoscope. Logistic regression models were employed to assess the independent associations of the most impactful variables.

### Primary outcome: diagnostic yield

We used a strict definition for diagnostic yield. We also calculated the sensitivity for diagnosis of malignancy in our cohort. Diagnostic yield (DY) was calculated as the rate of true positive (TP) plus true negative (TN) results for malignancy divided by total number of bronchoscopies. TN results were concluded based on the following criteria:

1. A specific benign (SPB) diagnosis was established (such as a granulomatous disease or a definitive infectious diagnosis).
2. If a nonspecific benign (NSB) result was reported at index bronchoscopy, such as atypical or inflammatory cells, then the case was assessed longitudinally and categorized as TN (NSB-TN) only if subsequent biopsy (either bronchoscopic, CT-guided or surgical biopsy) or imaging confirmed a nonmalignant diagnosis or follow up imaging after at least 12 months showed stability or resolution of the lesion.
3. The cases with normal lung/bronchi tissue reports from index bronchoscopy, and the cases in which a definitive diagnosis was not established because of lack of follow-up were counted as non-diagnostic bronchoscopy (ND). TN only included SBP and NSB-TN and not ND.

### Sensitivity calculation

Sensitivity was calculated as the percentage of proven malignant lesions by index bronchoscopy out of total cases of malignancy. Total cases of malignancy included those proven via bronchoscopy or other methods, plus any case that received empiric treatment for malignancy (without tissue diagnosis), plus any case that was lost to follow up.

### Secondary outcomes

1. Achievement of concentric rEBUS view.
2. Impact of addition of ultrathin bronchoscope on procedure outcomes.
3. Correlation of radiographic characteristics of lesions with diagnostic yield; size, location, distance from closest visceral pleural surface, PET characteristics, border characteristics in lesions ≤ 15 mm (smooth or irregular), bronchus sign, appearance of lesion.

### Variables

#### Concentric vs Eccentric rEBUS view

Concentric view is an ultrasonic image achieved when r-EBUS probe is positioned in the airways surrounded by the lesion, while eccentric view correlates with the images from airways adjacent to the lesion. Traditionally, concentric r-EBUS view correlates with higher diagnostic yield compared to eccentric view.

#### Lesion size

We measured the long axis (LA) diameter of the lesion in an axial plane of chest CT scan. We also measured a short axis which was defined as a perpendicular line crossing LA in the middle. Then the average of the long and the short axis was calculated.

#### Location

Based on the CT scan, the lesions in right upper lobe, right middle lobe, and left upper lobe were classified together compared to the lesions in right and left lower lobes.

#### Distance from closest visceral pleural surface

We used the distance from the center of the lesion to the closest visceral pleural surface (including fissure and mediastinal pleura) in the axial plane of the CT scan to objectively classify the location of the lesions relative to central airways. Due to lack of a standard definition for peripheral vs central lesion, we used this measurement to classify peripheral vs central lesions with a numerical value [32, 33]. The airways start branching from the lung hilum and extend to smaller airways in a semi-spherical pattern in each lobe. Visceral pleura marks the peripheral boundaries of each lobe, and the distance from the lesion to closest visceral pleural surface may best explain how peripheral the lesion is. This calculation takes into account the direction of airway extension in a spherical pattern. For example, a lesion located close to the midline of the body but close to the mediastinal pleura is called a peripheral lung lesion and may be challenging to reach via peripheral airways.

#### PET characteristics

The FDG avidity of the lesion reported by a PET/CT obtained 3 months before or after the index bronchoscopy was reported in numerical value.

#### Border characteristics

Based on the CT scan, the borders of the lesions were classified as smooth or irregular. Irregular border included the ones with spiculated or lobulated borders. Specifically in lesions ≤ 15 mm this characteristic was used to check for any possible correlation with diagnostic yield. We previously observed the smaller lesions with irregular borders to have a better chance of being detected by r-EBUS examination.

#### Bronchus sign

We reviewed the CT scan images in different planes and reported any visible air-filled airway which led to the targeted lesion. This is called a bronchus sign and previously has been reported to be associated with higher diagnostic yield [34, 35]. We examined whether the presence or lack of a bronchus sign had any impact on case selection by the bronchoscopist and if there was any correlation with diagnostic yield.

#### Nodule appearance

We reviewed the characteristics of the lesion in the CT scan images and based on a visual assessment, classified the lesions as solid, ground-glass, combined solid and ground-glass and cavitary.

### Case Selection

Patients who were referred to the Interventional Pulmonary service for evaluation of a lung lesion identified by chest CT scan were included in this study. Bronchoscopy was performed for lesions with diameter of 1 cm and above with estimated intermediate pretest probability for malignancy (5–65%). Sub-centimeter nodules were typically followed by surveillance imaging, however a few nodules smaller than 1 cm were selected for bronchoscopy because of high suspicion for malignancy and lack of alternative diagnostic approach. Patients with a high (> 65%) probability of malignancy were referred for surgical resection, but bronchoscopy was performed if they were too unstable for surgery or biopsy prior to surgery was preferred. Patients who were at non-reversible and high risk of respiratory or cardiovascular failure were excluded from bronchoscopy.

### Bronchoscopy procedure

CT scan images were reviewed before the procedure and were available for further review during the bronchoscopy if needed. In subjects with multiple pulmonary lesions, the decision about which and how many lesions to be sampled was made before bronchoscopy. The lung segment or segments containing the target lesion were identified based on airway anatomy and CT scan configuration. General anesthesia was administered by an anesthesiologist, with the majority of cases undergoing endotracheal intubation for the procedure. We advised our anesthesiologist against the use of paralytic agents. While deep sedation and general anesthesia were used, most of the cases were performed without apnea, even during the biopsy passes. The first part of the bronchoscopy included routine airway inspection to the segmental levels. Any patient with an endobronchial lesion was excluded from the study. We routinely used an Olympus BF-P190 bronchoscope (OD 4.2 mm, WC 2.0 mm) for airway inspection and clearance of airway secretions. After regular inspection, radial EBUS was employed to identify and localize the target lesion. Mediastinal staging using a linear array EBUS bronchoscope was performed when appropriate either before or after using rEBUS for a peripheral lung lesion.

A radial endobronchial ultrasound (rEBUS) probe (Olympus UM-S20–17S) with a wave frequency of 20MHz and OD 1.4 mm was inserted through the working channel of the flexible bronchoscope. Under 2D fluoroscopy the pre-identified lung segments which were assumed to contain the target lesion were examined. We rarely used a guide sheath for the rEBUS probe and no navigational platform was utilized. Every small branch of the airways in the relevant area was examined to find the lesion. This peripheral airway survey was continued until the best rEBUS view (preferably a concentric view) of the target was obtained. A rEBUS guide sheath could be used and worked as an extended channel when the tip of the scope was not parked close to the target, but it was rarely needed in our practice. After the ultrathin bronchoscope became available (3/4/2019), our bronchoscopy technique was modified. If a concentric rEBUS view was not obtained, if the target was not found at all, or if the distance from the tip of scope to the target was too long which could potentially cause redirection of the biopsy tools into wrong airways in subsequent passes, then the bronchoscope was switched to an ultrathin Olympus BF-MP190 bronchoscope (OD at tip 3.0 mm, WC 1.7 mm). The ultrathin bronchoscope could visualize smaller branches of the airways and get closer to the smaller lesions, leading to more concentric rEBUS view captures.

### Specimen collection

Once the optimal view of the lesion was identified, the rEBUS probe was removed while the tip of the bronchoscope remained in position. Trans-bronchial needle aspiration (TBNA) was performed using an Olympus PeriView FLEX or Olympus NA-1C-1 21-gauge needle. The needle specimens were reviewed by a cytotechnician during the procedure and depending on their feedback, further sampling vs. adjustment of the bronchoscope before further sampling was done. Then trans-bronchial biopsy was done using either a disposable Radial Jaw™ 4 Boston Scientific Pulmonary Standard Capacity 2.0-mm or Olympus FB-231D oval cup 1150mm X 2.0mm disposable biopsy forceps with thin scope. Olympus FB-433D disposable oval cup 1.5 mm biopsy forceps was used with ultrathin scope. Routinely 5 passes with needle and 5 passes with forceps were executed. Further sampling including trans-bronchial needle, forceps, brushing, and broncho alveolar lavage were performed at the discretion of the bronchoscopist.

### Post-bronchoscopy follow up

A biopsy that resulted in a specific diagnosis, either malignant or benign, was counted as a successful bronchoscopy (diagnostic). If no specific diagnosis was made based on the index bronchoscopy results, then the case was discussed among the multidisciplinary lung cancer team members which included an interventional pulmonologist, thoracic surgeon, medical and radiation oncologist, chest radiologist, pathologist, and nuclear medicine specialist. The following pathways were pursued based on the consensus recommendation:

#### Excisional biopsy or CT-guided biopsy

If it showed a similar result, then the bronchoscopy was counted as successful.

#### Surveillance imaging for 12 months or longer

If it showed stability or resolution of the lesion, then the bronchoscopy was counted as successful (excluded the cases in which cytology and pathology reports from the index bronchoscopy showed a normal lung or bronchial tissue).

#### Repeat bronchoscopy

If the lesion remained suspicious and risk of the above two pathways considered to outweigh their benefits, then a second diagnostic bronchoscopy was considered.

#### Empiric treatment

After exhaustion of the above procedures and if no diagnosis was made but the concern of the treating physicians was a malignant lesion, empiric treatment including stereotactic body radiation therapy (SBRT) was considered.

### Complications

Adverse event such as pneumothorax, significant bleeding or any other significant event during bronchoscopy which required escalation of care such as hospital admission of an out-patient procedure or ICU transfer of a patient admitted on medical floor were documented. Every bronchoscopy was followed by a portable chest x-ray to rule out pneumothorax. Chest ultrasonography was performed in some cases to exclude pneumothorax. The number of cases requiring intervention, such as chest tube placement, was reported.

### Statistical methods

Continuous variables were compared between groups using the Wilcoxon rank sum test. The chi-square or Fisher’s exact test was used to compare categorical variables. Logistic regression models were also fit to the data to assess the independent associations of concentric rEBUS view, solid appearance, upper/middle lobe location, and larger nodule size with successful bronchoscopy in the group after the ultrathin scope became available. A two-sided *P* < 0.05 was considered statistically significant. All analyses were conducted using SAS 9.4.

## Results

A total of 322 patients underwent diagnostic bronchoscopy for evaluation of peripheral lung lesions. Mean age of patients was 66 (SD 12.7); 58% were female. The study population consisted of a mixture of different races including 29% African American and 36% Hispanic. Lung lesion sizes ranged from 0.7 cm to 9.4 cm in the long axis diameter in the axial plane of CT scan. The median long axis diameter of lesions was 2.2 cm. We classified nodules with diameter of less than 3 cm into four groups with 5 mm intervals to be able to detect any differences in diagnostic yield and correlate more precisely with other potential variables (Table 1). The median distance from the center of the lesion to the visceral pleural surface was 1.9 cm.

**Table 1:** Lung lesions classification based on long axis diameter in axial plane of CT scan

	Lesion Size (axial diameter) (cm)								
	< 1 cm	1–1.5 cm	1.51–2 cm	2.1–2.5 cm	2.51–3 cm	3.1–4 cm	4.1–5 cm	> 5 cm	Total
Number of cases (%)	13 (4.04)	71 (22.05)	59 (18.32)	34 (10.56)	38 (11.80)	48 (14.91)	17 (5.28)	42 (13.04)	322 (100.00)

Overall diagnostic yield (DY) was 76.6%. DY before and after the employment of the ultrathin scope in practice was 73.6% and 81.3% respectively (*p* = 0.11). The sensitivity for detecting malignancy increased from 60.5% to 74.7% (*p* = 0.033) (Table 2). The ultrathin bronchoscope was used in 64 of 123 cases since it became available. 78% (50/64) of those cases had lesions with diameter equal to or smaller than 2 cm. The ultrathin scope was rarely needed for lesions above 3 cm.

**Table 2:** Comparing sensitivity and diagnostic yield before and after ultrathin scope was available

	Before ultrathin scope was available	After ultrathin scope was available	*P*-value
Sensitivity	78/129 = 60.5%	62/83 = 74.7%	0.033
Diagnostic yield	145/197 = 73.6%	100/123 = 81.3%	0.11

We compared the cases before and after employment of ultrathin scopes (pre- and post-era). Table 3 compares demographics, characteristics of lesions, and bronchoscopic findings in the two groups. More concentric rEBUS views were detected in post-era (93% vs. 78%, *p* < 0.001). Nodule sizes were similar in the two eras. The median long axis diameter was 2.3 cm in pre-era and 2.1 cm in post-era (*p* = 0.708). There were no differences in radiographic appearance, border characteristic or location of the lesions between the two groups. Bronchus sign was seen in 99/199 (50%) of patients in pre-era group compared to 45/123 (37%) of patients in post-era group (*p* = 0.021). The distance from visceral pleura to center of lesion was significantly different between the two groups. The median distance in the pre-era was 2 cm and in post-era was 1.5 cm (*p* = 0.018). This represents more peripheral lesions in post-era compared to pre-era (Table 3).

**Table 3:** Bivariate associations with Era

Variable	Available numbers	Overall *N* = 322	Pre *N* = 199	Post *N* = 123	*P*-Value
Radial EBUS view					
Non-Concentric	322	52 (16.15%)	43 (21.61%)	9 (7.32%)	** < .001**
Concentric		270 (83.85%)	156 (78.39%)	114 (92.68%)	
Appearance					
Solid	322	237 (73.60%)	146 (73.37%)	91 (73.98%)	0.903
Non-Solid		85 (26.40%)	53 (26.63%)	32 (26.02%)	
Border characteristic					
Regular	321	56 (17.45%)	36 (18.09%)	20 (16.39%)	0.697
Irregular		265 (82.55%)	163 (81.91%)	102 (83.61%)	
Location					
Upper or middle lobe	321	228 (71.03%)	141 (71.21%)	87 (70.73%)	0.927
Lower lobe		93 (28.97%)	57 (28.79%)	36 (29.27%)	
Bronchus sign					
Absent	322	178 (55.28%)	100 (50.25%)	78 (63.41%)	**0.021**
Present		144 (44.72%)	99 (49.75%)	45 (36.59%))	
Sex					
Female	322	187 (58.07%)	128 (64.32%)	59 (47.97%)	**0.004**
Male		135 (41.93%)	71 (35.68%)	64 (52.03%)	
Nodule diameter size- long axis (cm)	322	2.2 (1.5, 3.5)	2.3 (1.5, 3.6)	2.2 (1.5, 3.5)	0.708
Nodule diameter size—Average (cm)	322	1.95 (1.3, 3.25)	1.95 (1.25, 3.25)	1.95 (1.35, 3.3)	0.882
Distance from visceral pleura (cm)	322	1.9 (1.1, 2.6)	2 (1.3, 2.9)	1.5 (0.9, 2.2)	**0.018**
BMI	318	26.11 (21.78, 30.81)	26.6 (21.68, 31.54)	25.07 (21.8, 29.18)	0.100
FDG avidity	234	5 (2.9, 8.9)	5 (3, 8.7)	5 (2.9, 9.1)	0.901
Age	322	69 (58, 75)	68 (58, 74)	70 (57, 76)	0.398

The bold emphasize the statistical significance

In the post-era group, the concentric r-EBUS view, appearance of the lesion and the long axis diameter of the lesion in axial CT scan plane were associated with successful bronchoscopy. Border characteristics, location, bronchus sign, average of long and short axes sizes, distance from pleura, BMI, and FDG avidity were not associated with successful bronchoscopy (Table 4). In the pre-era group, the concentric r-EBUS view, border characteristics, bronchus sign, nodule diameter both the long axis and average sizes, distance from pleura and BMI were associated with successful bronchoscopy, while the appearance of the lesion and FDG avidity were not (Table 5).

**Table 4:** Post-Era only: Bivariate associations with diagnostic yield success

Variable	Level	Available numbers	Not success *N* = 23	Success *N* = 100	*P*-Value
Radial EBUS view	Non-concentric	123	6 (66.67%)	3 (33.33%)	**0.001**
	Concentric		17 (14.91%)	97 (85.09%)	
Appearance	Solid	123	12 (13.19%)	79 (86.81%)	**0.008**
	Non-solid		11 (34.38%)	21 (65.63%)	
Border characteristic	Regular	122	5 (25.00%)	15 ((75.00%)	0.531
	Irregular		18 (17.65%)	84 (82.35%)	
Location	Upper or middle lobe	123	13 (14.94%))	74 (85.06%)	0.097
	Lower lobe		10 (27.78%)	26 (72.22%)	
Bronchus sign	Absent	123	18 (23.08%)	60 (76.92%)	0.101
	Present		5 (11.11%)	40 (88.89%)	
Sex	Female	123	12 (20.34%)	47 (79.66%)	0.654
	Male		11 (17.19%)	53 (82.81%)	
Nodule diameter size- long axis (cm)		123	1.8 (1.4, 2.2)	2.3 (1.6, 3.8)	**0.047**
Nodule diameter size- Average of long and short axes (cm)		123	1.75 (1.3, 2.1)	2.1 (1.35, 3.45)	0.095
Distance from visceral pleura (cm)		123	1.1 (0.7, 2.1)	1.6 (1.1, 2.3)	0.133
BMI		119	22.49 (18.55, 29.39)	25.17 (22.33, 28.94)	0.200
FDG avidity		93	3.6 (2.7, 5.6)	5.8 (2.9, 9.9)	0.061
Age		123	72 (62, 80)	69.5 (57, 76)	0.237

The bold emphasize the statistical significance

**Table 5:** Pre-Era only: Bivariate associations with diagnostic yield success

Variable	Level	Available numbers	Not success *N* = 23	Success *N* = 100	*P*-value
Radial EBUS view	Non-concentric	197	28 (68.29%)	13 (31.71%)	** < 0.001**
	Concentric		24 (15.38%)	132 (84.62%)	
Appearance	Solid	197	38 (26.21%)	107 (73.79%)	0.920
	Non-solid		14 (26.92%)	38 (73.08%)	
Border characteristic	Regular	197	17 (47.22%)	19 (52.78%)	**0.002**
	Irregular		35 (21.74%)	126 (78.26%)	
Location	Upper or middle lobe	196	37 (26.43%)	103 (73.57%)	0.959
	Lower lobe		15 (26.79%)	41 (73.21%)	
Bronchus sign	Absent	197	39 (39.39%)	60 (60.61%)	** < 0.001**
	Present		13 (13.3%)	85 (86.7%)	
Sex	Female	197	38 (30.16%)	88 (69.84%)	0.110
	Male		14 (19.72%)	57 (80.28%)	
Nodule diameter size- long axis (cm)		197	1.5 (1.2, 2.3)	2.8 (1.8, 4)	** < 0.001**
Nodule diameter size- Average of long and short axes (cm)		197	1.3 (1.025, 1.9)	2.35 (1.5, 3.65)	** < 0.001**
Distance from visceral pleura (cm)		197	15.88 (10.5, 24)	21 (14, 29.7)	**0.045**
BMI		197	28.93 (22.29, 33.00)	26.4 (21.67, 30.13)	**0.043**
FDG avidity		139	4.1 (2.5, 8)	5.9 (3.4, 9)	0.169
Age		197	64 (57.5, 74.5)	69 (58, 74)	0.401

The bold emphasize the statistical significance

The multivariate regression model in post-era found the concentric rEBUS image acquisition during bronchoscopy, solid appearance in CT scan, upper and middle lobe location of nodule and larger nodule size to be independent predictors of diagnostic yield (Table 6).

**Table 6:** Multivariable logistic regression modeling of predictors of diagnostic yield success in post-era only

Odds ratio estimates				
Effect	Point estimate	95% Wald confidence limits		*P*-value
Concentric vs non-concentric r-EBUS View	7.859	1.496	41.294	0.0149
Solid vs non-solid appearance	3.211	1.043	9.886	0.0421
Upper and middle vs lower lobe location	6.092	1.829	20.297	0.0033
Nodule size in long axis	1.874	1.085	3.235	0.0242

Among the diagnoses of malignancy, non-small cell lung cancer was predominant (72.41%), followed by metastatic lesions (12.81%). Sarcoidosis was the most common specific benign diagnosis (28.0%) followed by fungal infection (20.0%) (Table 7 and 8).

**Table 7:** Malignant Diagnosis

Malignant diagnosis	Overall	Pre	Post
NSCLC	147 (72.41%)	90 (73.17%)	57 (71.25%)
SCLC	4 (1.97%)	2 (1.63%)	2 (2.50%)
Lymphoma	6 (2.96%)	2 (1.63%)	4 (5.00%)
Others/Neuroendocrine/Carcinoid/ etc.	5 (2.46%)	3 (2.44%)	2 (2.50%)
Metastases	26 (12.81%)	15 (12.20%)	11 (13.75%)
Unclassified	15 (7.39%)	11 (8.94%)	4 (5.00%)

**Table 8:** Benign Diagnosis

Benign diagnosis	Overall	Pre	Post
Sarcoidosis	14 (28.00%)	10 (30.30%)	4 (23.53%)
Fungal Infection	10 (20.00%)	4 (12.12%)	6 (35.29%)
Other infections	18 (36.00%)	14 (42.42%)	4 (23.53%)
Hamartoma	3 (6.00%)	2 (6.06%)	1 (5.88%)
Unclassified	5 (10.00%)	3 (9.09%)	2 (11.76%)

In the pre-era, the average number of needle passes was 3.89 and number of forceps was 3.18. In the post-era, the average number of needle passes was 4.62 and number of forceps was 4.03. Additional testing on malignant lesions was performed more in post-era likely from the oncology practice change over time which required more tissue for molecular analysis (EGFR, ALK, ROS, etc.) and PD-L1. Forty-one out of 53 cases with malignant results (77.4%) had sufficient material for molecular testing; and similarly, 77.4% had sufficient material for PD-L1 testing.

### Complications

Three patients developed pneumothorax, two of them were in pre-era. All three patients were treated with chest tube insertion. The pneumothorax resolved in 1–2 days and no further treatment was needed. Massive bleeding which required escalation of care was not observed. There were 5 cases of airway bleeding which required either local thrombin injection or temporary balloon occlusion. Three of the cases occurred in the pre-era and two in the post-era.

## Discussion

We conducted a retrospective study of diagnostic bronchoscopy for peripheral lung lesions in a tertiary care university hospital. We employed conventional tools including radial EBUS and 2D fluoroscopy without any navigational system. After addition of ultrathin bronchoscope to our basic tools, our sensitivity for detection of malignancy increased from 60.5% to 74.7%. In this era our diagnostic yield (DY) based on a strict definition [36] was 81.3% and the concentric radial EBUS views increased to 92.7%. Our results compare favorably with the highest levels of success currently reported in literature using other navigational and robotic systems.

In the last few years different bronchoscopic techniques have been developed, but well-designed comparison studies are lacking, and the optimum technology with affordable costs is yet to be defined [8]. The benefits of some of the prior commonly used modalities are now being questioned [37]. Robotic bronchoscopy platforms have increased DY in some institutions with reported standalone yield of approximately 80% [13–16]. By adding 3D imaging during bronchoscopy the yield has improved further [22–26]. With increasing incidence of lung nodules, the consequent cost of diagnostic procedures is expected to become a prominent health-care system burden. The ultrathin bronchoscope has been reported to be a useful tool for diagnosis of peripheral lung lesions but has not yet been commonly adapted in clinical practice [27–31]. Our study may justify the combination of ultrathin bronchoscopy and other basic tools including rEBUS and 2D fluoroscopy as an alternative to more expensive techniques such as robotic bronchoscopy where the costs of the system and consumables may outweigh the small increase in diagnostic yield of most of the lung nodule cases.

**Fig. 1:**
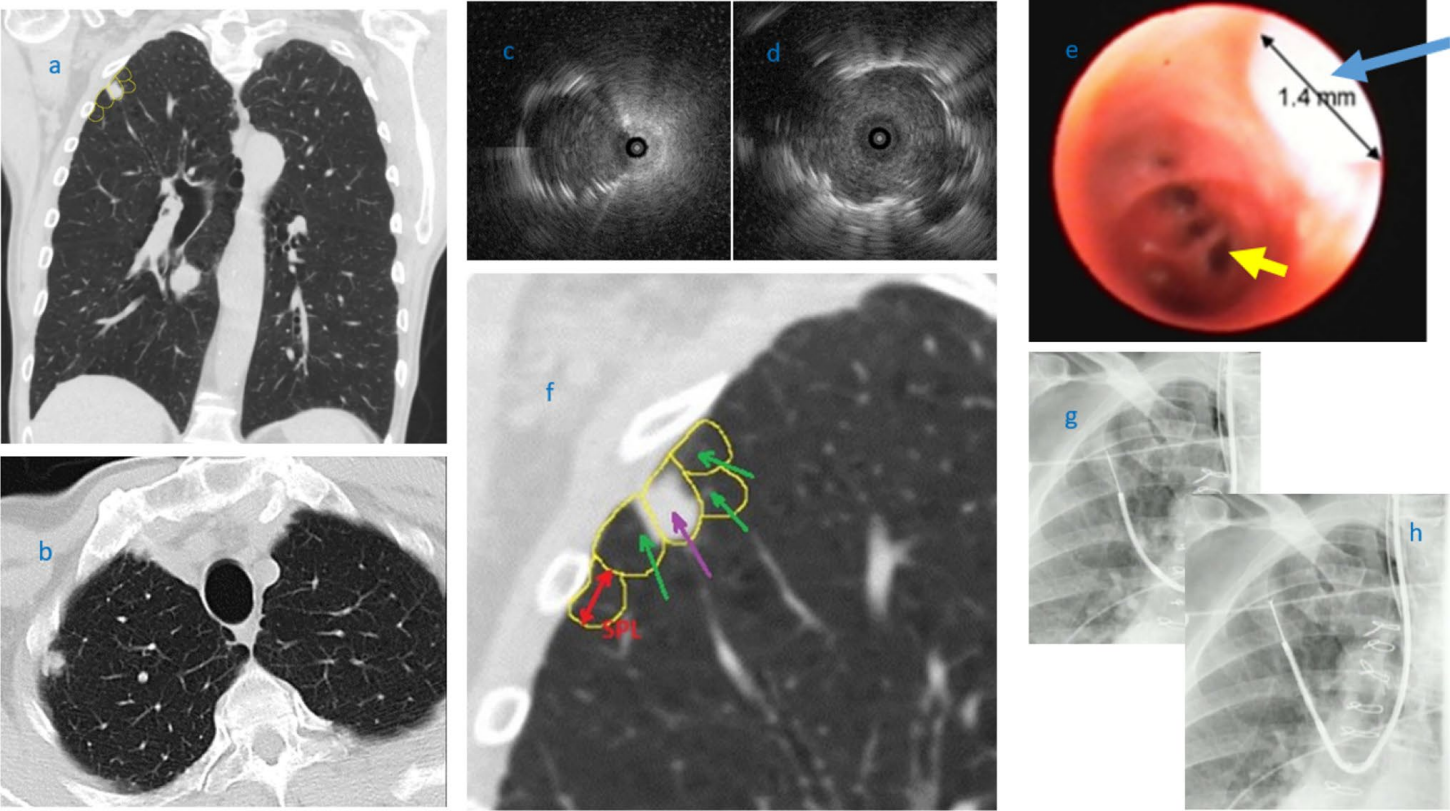
Representative coronal and axial planes view of CT scan, showing a small apical right upper lobe nodule (**a**, **b**). Eccentric radial EBUS (rEBUS) view of the same nodule captured by a 4.2-mm thin bronchoscope (**c**). Concentric rEBUS view of the same nodule captured by a 3.0-mm ultrathin bronchoscope (**d**). Bronchoscopic view of multiple lobular bronchioles (the yellow arrow pointing to one of them) and the tip of a rEBUS probe with 1.4-mm diameter (blue arrow) captured by ultrathin bronchoscope (**e**). Few secondary pulmonary lobules (SPL) delineated by yellow line around the lung nodule. The green arrows show the SPLs which provide eccentric rEBUS view, and the purple arrow shows the SPL which provides concentric rEBUS view. (**f**) Fluoroscopic images of transbronchial needle aspiration and forceps biopsy via ultrathin bronchoscope (**g**, **h**). Cytology and histopathology results showed squamous cell lung carcinoma

The high number of concentric rEBUS views is explained by the ability of the ultrathin scope to maneuver in airways as small as 3 mm in diameter and visualize more peripheral branches and reach closer to the target in comparison with a conventional bronchoscope. We reviewed the effect of the lesion size on diagnostic yield based on the classification shown in Table 1. The high diagnostic yield was maintained close to 80% until the nodule size dropped less than 1.5 cm (Table 9). This can be explained by the maneuverability and enhanced visualization of small airways close to secondary pulmonary lobule (SPL) which are known as the smallest morphologic and functional units of the lung. Each SPL has an average diameter of about 10–25 mm, separated by interlobular septae. They contain lobular bronchioles (lumen size of about 1.0 mm) and terminal bronchioles (lumen size of about 0.6 mm) at the end of the tracheobronchial tree (Fig. 1) [38–40]. We have encountered many instances in which the size of airways in front of the camera were smaller than 1 mm, representing the lobular branches of the airway tree and close to terminal bronchiole. On many occasions the tip of the scope reaches the lesions or even lands inside them. The scope can be used as a conduit or extended channel to pass biopsy tools inside the nodule. On a routine basis, we obtain multiple rEBUS views of the lesion from parallel small airways surrounding the lesion; some are eccentric, some concentric. We can then choose the best airway through which to pass diagnostic tools into the lesion. Figure 1 shows a case in which the ultrathin scope allowed identification of an airway which led to the center of lesion and changed the eccentric view to concentric.

**Table 9:** Post -Era only: Association between Size and Diagnostic Yield

		Lesion size (cm)							
	< 1 cm	1–1.5 cm	1.51–2 cm	2.1–2.5 cm	2.51–3 cm	3.1–4 cm	4.1–5 cm	> 5 cm	Total
Number of cases (%)	2 (1.63)	27 (21.95)	29 (23.58)	13 (10.57)	13 (10.57)	20 (16.26)	8 (6.50)	11 (8.94)	123 (100.00)
Successful result *n* (%)	2 (100.00)	19 (70.37)	23 (79.31)	8 (61.54)	11 (84.62)	18 (90.00)	8 (100.00)	11 (100.00)	100 (81.30)

Multivariate regression analysis in post-era found that concentric rEBUS view, solid appearance, upper/middle lobe location and larger nodule size were independent predictors of diagnostic yield (Table 6). This information may be applied in clinical practice when selecting patients for bronchoscopy and can help predict outcomes. Concentric rEBUS view is known to be associated with increased diagnostic yield and this was also confirmed in our study (odds Ratio 7.9, *p* = 0.01). The high rate of concentric rEBUS view (92.7%) when using ultrathin bronchoscopy will give more confidence to achieve a satisfactory outcome. Solid appearance of nodules on CT scan was another predictor of a better outcome (odds Ratio 3.2, *p* = 0.04). In non-solid lesions, we consider adding other modalities for real time confirmation that are more sensitive than ultrasound in detecting ground glass opacities. Location of the lesion in upper lobes or right middle lobe of lung were found to be associated with higher diagnostic yield (odds Ratio 6.1, *p* = 0.003). One possible explanation is the fact that upper and middle lobes have lower rate of atelectasis during bronchoscopy which might negatively impact navigation toward the lesion. To avoid atelectasis, we apply higher positive end expiratory pressure (PEEP) and low Fio2 during mechanical ventilation. We have also applied decubitus positioning of the patient when the lesion is in dependent areas with a high likelihood of atelectasis. We found this positioning of patient to be feasible as we were not dependent on any navigational systems which require the patient to be in a supine position. Another possible explanation is the higher incidence of lung cancer in those lobes compared to lower lobes. Larger lesions were associated with increased DY (OR 1.88, *p* = 0.024). This is consistent with previously published data, that DY is inversely associated with smaller size, however the point estimate for size was smaller than the other variables in predicting the success rate.

In the post-era group the lesions were more peripheral as measured by the distance of the center of lesions to the closest visceral pleural surface. Interestingly, a peripheral lesion was not associated with lower success, adding to the benefits of the ultrathin bronchoscope, likely due to its ability to better reach the lung periphery. Additionally, there was a low rate of bronchus sign in the post-era group (36.6%) but it did not affect diagnostic yield (Tables 3 and 4). These findings confirm that thin and ultrathin bronchoscopy can navigate through small airways that are not detected by CT scan, and bronchoscopists should not be discouraged from performing bronchoscopy for lesions without a bronchus sign. The better reach to peripheral lesions by thin and ultrathin bronchoscopes also makes them useful conduits for passing tools directly into lesions. Having a constant eye on the target while sampling allows maintenance of the tools in stable position for repeated sampling. This enhanced bronchoscopic visualization can also explain the lower risk of complications as it decreased injury to the surrounding structures including pleural surface, vessels, and normal lung parenchyma. In our prior study, we used a mobile 3D fluoroscopy system and were able to externally validate the precise positioning of the tip of the ultrathin scope relative to small lung lesions for confirmation of tools in target [22]. Ultrathin bronchoscopy has reached a satisfactory point in terms of tools in target confirmation by giving high concentric rEBUS views and stability during multiple sampling. The other challenge that needs improvement is biopsy tool. Adding new tools to improve the quantity and quality of specimens, such as cryo biopsy, may further improve DY [41]. Other adjunct technologies such as 3D imaging may improve micro-navigation around sub-centimeter nodules and other ground glass lesions.

We calculated the average of long and short axes in axial plane CT scan to see if the geometrical shape of a lesion has any effect on bronchoscopy yield. While the long axis was associated with diagnostic yield, the average of the long and short axes was not in post-era (Table 4). This suggests that the yield of biopsy is not affected by the shape of the lesion and rounded vs elongated lesions are not different in terms of bronchoscopy outcomes. A likely explanation is that elongated lesions potentially have a higher chance to reach to airways and thus be visualized during bronchoscopy. FDG avidity showed some trends toward increased DY in post-era bivariate analysis. Location and border characteristics of nodules were not associated with DY, and this remained the same when adjusted for smaller size nodules even less than 1.5 cm (Tables 4 and 10).

**Table 10:** Association between border characteristic and diagnostic yield in subgroup of nodules with long axis diameter of < 1.5 cm in the post-era group

Border characteristic	Overall *N* = 29	Non diagnostic *N* = 8	Diagnostic *N* = 21	*P*-value
Regular	11 (37.93%)	1 (12.50%)	10 (47.62%)	0.110
Irregular	18 (62.07%)	7 (87.50%)	11 (52.38%)	

Compared to post-era group, in the pre-era group before utilization of the ultrathin bronchoscope, aside from the concentric rEBUS view and the long axis diameter, the following variables could have an impact on diagnostic yield: border characteristic of lesion, bronchus sign, average of long and short axis diamater, distance from pleura and BMI. However, the radiological appearance of the lesion was not associated with the DY in this group compared to post-era group (Table 5).

We reviewed the number of passes of transbronchial needle aspiration (TBNA) and transbronchial forceps biopsy (TBBx) in both pre- and post-era. On average the number of passes on both sampling methods increased (TBNA from 3.89 to 4.62 and TBBx from 3.18 to 4.03). It is not clear if the number of passes between the two groups had any effect on diagnostic yield. It is possible that increasing demand over time for more tissue to assess for molecular profile and PD-L1 in addition to what is needed for tissue diagnosis have caused the higher number of sampling passes. In patients with a confirmed diagnosis of malignancy in post-era, 77.4% had sufficient material for molecular testing (EGFR, ALK, ROS, etc.) and the same for PD-L1 testing. We may conclude that the ultrathin bronchoscope along with its current sampling tools provide enough tissue for the ever-increasing demand of advanced testing in oncology practice.

## Limitations of the study

Our study is a retrospective review of the practice in a single academic medical center with expertise in peripheral airway bronchoscopy. Therefore, there is a limitation to generalize its findings to a broad practice of bronchoscopy in different academic and community settings. We explained our bronchoscopic findings based on the anatomical relationship and hope this illustration of the concepts helps for adaptation of ultrathin bronchoscopy. We used general anesthesia via an endotracheal tube in most cases. This method may need to be studied further in centers where general anesthesia is not available. Another limitation is the longitudinal cohort comparison, which has a built-in timeline bias. However, in this single center study, the practice pattern did not have any obvious or significant change over time except for the addition of the ultrathin bronchoscope.

## Conclusion

The combination of basic bronchoscopy tools with rEBUS and the ultrathin bronchoscope is promising for the improvement of diagnosis of lung cancer in early stages when lesions are small. The high DY associated with thin and ultrathin bronchoscopes is attributed to the advantages provided by improved visualization of small peripheral airways with simultaneous rEBUS confirmation and sampling. The precision in reaching peripheral lung nodules, high diagnostic yield, and safety profile along with low-cost render ultrathin bronchoscopy a potentially reliable method for universal adaptation.
